# *In vitro* Metabolomic Approaches to Investigating the Potential Biological Effects of Phenolic Compounds: An Update

**DOI:** 10.1016/j.gpb.2016.12.007

**Published:** 2017-05-24

**Authors:** Úrsula Catalán, Laura Barrubés, Rosa Maria Valls, Rosa Solà, Laura Rubió

**Affiliations:** 1 Functional Nutrition, Oxidation and Cardiovascular Diseases Group (NFOC-Salut), Technological Center of Nutrition and Health (CTNS), Institut d’Investigació Sanitària Pere Virgili (IISPV), Faculty of Medicine and Health Sciences, Universitat Rovira i Virgili, Reus 43201, Spain; 2 Food Technology Department, Universitat de Lleida-AGROTECNIO Center, Lleida 25198, Spain

**Keywords:** Cell-based, Metabolomics, Phenolic compounds, Mechanism of action, Disease prevention

## Abstract

Dietary **phenolic compounds** (PCs) have been receiving interest for their presumed roles in **disease prevention**. However, there is a lack of studies on the underlying molecular mechanisms. In this regard, *in vitro***metabolomic** approaches are suitable for the investigation of the molecular changes in response to PC exposure. Up to date, the biological effects of PCs have only been examined for PCs from rosemary (*Rosmarinus officinalis*), olive oil, and resveratrol using **cell-based** metabolomic approach, although transcriptomic and/or proteomic studies have also been conducted in the same *in vitro* cell experiment in some cases. Our integral analysis of the reviewed studies suggest that PCs may be involved not only in basic cellular processes or macro- and micro-nutrient metabolism, but also in specific metabolic pathways that have been thoroughly investigated. These modulated pathways could have a clinical impact on neurodegenerative diseases, type 2 diabetes, cancer, and cardiovascular diseases. In conclusion, the *in vitro* metabolomic approaches provide additional information of the molecular mechanisms involved in disease risk reduction of dietary PCs. In order to elucidate the mechanisms of action of PCs, more metabolomic cell-based studies are needed and testing the physiological conjugated forms of PCs in these cell systems could be of special interest.

## Introduction

Phenolic compounds (PCs) are characterized by a chemical structure of hydroxylated aromatic ring(s). They are ubiquitously present in plants as secondary metabolites [1] and represent the most quantitative phytochemicals supplied by the diet [2]. So far,∼8000 PCs have been identified, which can be classified into different subclasses according to the number of aromatic rings in their structure, the elements that bind the rings with each other, and the substituents linked to the rings. Accordingly, four main families can be identified, namely phenolic acids, flavonoids, stilbenes, and lignans [3].

PC consumption is estimated at about 1564.56 mg of gallic acid equivalent/person/day [4]. The most important nutritional sources of PCs are fruits and vegetables, green and black tea, red wine, coffee, cocoa, and extra virgin olive oil [5]. Likewise, herbs, spices, nuts, and algae are potentially important sources of PCs as well, depending on culinary habits [6]. During the absorption and metabolism process, PCs are also subjected to changes and their bioactivity can consequently be modified [7].

PCs have been widely examined in humans, animals, and *in vitro* studies [1]. Currently, there is an increasing interest in exploring the practical applications of PCs focusing on their health-promoting and disease-preventing properties against type 2 diabetes mellitus (T2DM) [8], cardiovascular diseases (CVDs) [9], neurodegenerative diseases [10], or cancer [11]. However, mechanistic studies are still lacking.

In this context, metabolomics, transcriptomics, and proteomics may contribute to elucidating the molecular targets of PCs [12]. Each omics technology, alone or all together, is offering the researchers the possibility of linking food with health and diseases. The comprehensive vision to improve consumer’s well-being and knowledge using and integrating advanced omics platforms is called ‘foodomics approach’ [13]. Therefore, foodomics might provide a huge quantity of data about the pathways which PCs could be involved in and the information extracted could be translated to prevent, improve the time course, or even reverse the disease progression [13].

In particular, the metabolomic approach is becoming one of the mostly-used tools currently for investigating changes in the metabolome of biological systems, providing information on the small molecules in cells or tissues and defining the metabolic signature, thus leading to enhanced understanding of the disease mechanisms [14], [15]. Metabolomics assesses the final downstream products of gene transcription and is a good reflection of the biological system operation, namely its phenotype [16], [17]. The potential applications of metabolomic profiling techniques for evaluating bioactivity and the possible nutraceutical properties of diverse foods and/or bioactive compounds have created unprecedented opportunities.

Single PCs might have numerous molecular targets and subsequent physiological effects, which might not be easily addressed through classic target biomarker approach. As a result, the conventional use of particular biomarkers as indicators of health/disease must be replaced by comprehensive metabolic profiling. For instance, the conventional approach to studying CVD development in *in vitro* cellular studies is mostly based on some specific disease molecules (inflammation, endothelial dysfunction, or thrombosis, *etc*.) at the protein and/or RNA level, which only allows a partial understanding of the mechanisms of action [18]. However, *in vitro* metabolomics can focus on a small number of components, providing practical global knowledge about the metabolic pathways that PCs are involved in [19].

Beyond the common biofluids analyzed in metabolomics (plasma, urine, and fecal extracts), cell cultures offer the possibility for studying food ingredients or bioactive compounds (as well as new drugs or xenobiotics) in human biological materials more economically. *In vitro* studies can also offer benefits in terms of ethical considerations and can result in faster development [14].

Despite that, very few comprehensive studies have been performed that focus on the research of the molecular changes in response to PC exposure. Thus, in the present update, we aimed to integrate all the available bibliographic information related to the changes in the endogenous metabolite profile induced by the external stimuli of PCs based on an *in vitro* metabolomic approach. To our knowledge, this is the first time that underlying mechanisms of PCs in the disease risk reduction resulting from *in vitro* metabolic expression data are discussed.

## Search strategy for the bibliographic review

The bibliographic review was carried out in PubMed (http://www.ncbi.nlm.nih.gov/pubmed) and Scopus (http://www.scopus.com). The following terms were used for the search: ‘polyphenols’, ‘phenolic compounds’, ‘cells’, ‘mechanism of action’, and ‘metabolomics’.

**Figure 1:**
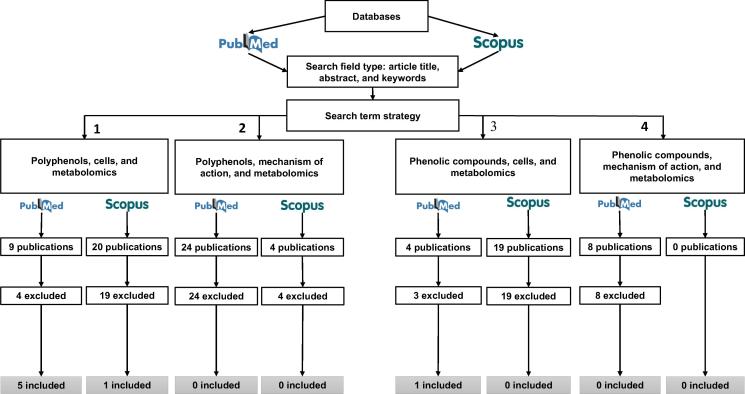
**Flowchart of the search strategy** Bibliographic review was carried out in PubMed and Scopus scientific databases until January 2016. The following terms were used for the search: ‘polyphenols’, ‘phenolic compounds’, ‘cells’, ‘mechanism of action’ and ‘metabolomics’. Reviews and studies evaluating metabolomics on plants, mice, rats or human samples were excluded and only cell-based studies were retained.

The search strategy flowchart is shown in Figure 1. Using the first search strategy, we found 29 publications, including 9 in PubMed and 20 in Scopus, of which four were excluded from PubMed search for being reviews, for not being a cell culture experiment, or for evaluating metabolomics of the tested food compounds. Nineteen publications were excluded from Scopus search for matching publications found in the PubMed search, for being reviews, or for evaluating metabolomics in human or hamster biofluids, or plants.

Using the second search strategy, we found 28 publications (24 in PubMed and 4 in Scopus), of which 24 were excluded from PubMed search for matching publications found in the first search strategy, for being editorials or reviews, or for evaluating metabolomics on plants, mice, rats, or human samples. Four publications were excluded from Scopus search for matching those found using the first strategy and by PubMed search using the second strategy, for being a review and for conducting metabolomics in human biofluids.

Using the third search strategy, we found 23 publications (4 in PubMed and 19 in Scopus), of which three were excluded from PubMed search for evaluating metabolomics on plants. Nineteen publications from Scopus search were excluded for matching publications found using the first two strategies and by PubMed search using the third strategy, for corresponding to reviews, for being a book chapter, or for evaluating metabolomics on plants and fungus.

Using the fourth search strategy, we found 8 publications (8 in PubMed and 0 in Scopus), of which eight were excluded from PubMed search for being the same publications found using the first three strategies, for being a review, or for evaluating metabolomics on plants, mice, or pigs. No publications were found in Scopus. Finally, seven *in vitro* metabolomic studies based on the effects of several PCs were selected [20], [21], [22], [23], [24], [25], [26]. Table 1 shows the main characteristics of the selected publications. Among these, five publications provided information regarding rosemary phenolic extract on human colon cancer cells HT-29 [20], [21], [23], [24] and human erythroleukemia cell lines K-562 [22]. Valdés et al. [20] tested the contribution of carnosic acid, the major PC in rosemary, individually instead of using the whole phenolic extract. The other two studies examined the effect of a phenolic extract from virgin olive oil containing secoiridoids, phenolic alcohols, and lignans on colon cancer cells SW-480 and HT-29 [26], and the effect of resveratrol on human hepatocellular carcinoma cells HepG2 [25].

**Table 1:** ***In vitro* foodomic studies evaluating the effects of PCs reviewed in this update**

**Cell types**	**PCs and tested concentration**	**Type of omics analysis**	**Method**	**Databases used for metabolite identification**	**Expression changes**	**Biological functions and pathways**	**Ref.**
Human colon cancer cells: HT29	Rosemary phenolic extract (carnosic acid: 256.0 µg/mg); 9.9 µg/ml	Transcriptomics	Microarray; RT-qPCR	—	281 DEGs out of 341 identified genes	Methylglyoxal degradation; dopamine, tryptophan, melatonin, noradrenaline, and serotonin degradation; ethanol degradation; bile acid and retinoate biosynthesis; LPS/IL-1-mediated inhibition of RXR function; transport of molecules; metabolism of terpenoid; metabolism of ROS; polyamine metabolism	[20]

		Metabolomics (nontargeted)	CE-MS and HILIC/UPLC-MS	HMD, METLIN, and KEGG	21 DEMs	Glutathione metabolism; polyamine metabolism	

	Rosemary phenolic extract (carnosol: 226.4 µg/mg; carnosic acid: 151.5 µg/mg); 10 µM (total rosemary phenols)	Transcriptomics	Microarray; RT-qPCR	—	1308 DEGs	Cellular development; cell death; cellular growth and proliferation; cell cycle; glutamate and glutathione metabolism	[21]

		Proteomics	MALDI-TOF/TOF-MS	—	17 DEPs	Cell proliferation; cell adhesion; cell migration	

	Rosemary phenolic extract (carnosol: 226.4 µg/mg; carnosic acid: 151.5 µg/mg); 10 µM (total rosemary phenols)	Metabolomics (nontargeted)	CE-MS, RP/UPLC-MS, and HILIC/UPLC-MS	HMD, METLIN, KEGG compound, and PubChem	65 DEMs	Glutathione metabolism; urea cycle and metabolism of amino groups; polyamines homeostasis; cell proliferation and viability	[23]

	Rosemary phenolic extract; 10 µM	Metabolomics (nontargeted)	CE-ESI-TOF MS	HMD, METLIN, KEGG compound, and PubChem	44 metabolites identified	Glycan biosynthesis and metabolism; organismal systems; excretory system; neuroactive ligand-receptor interaction; amino, carbohydrate, lipid, nucleotide, energy, cofactor, and vitamin metabolism; environmental and genetic information processing	[24]

Human colon cancer cells: SW480 and HT29	Extra virgin olive oil extract (decarboxymethyl oleuropein aglycon: 238.4 mg/kg); 0.01%–0.1%	Metabolomics (nontargeted)	nanoLC-ESI-TOF-MS	—	20 metabolites were identified in cells and culture medium	Uptake and metabolism of polyphenols; cell-cycle metabolism	[26]

Human hepatocellular liver carcinoma cells: HepG2	Resveratrol; 40 µM	Metabolomics (nontargeted)	^1^H NMR spectroscopy	—	9 DEMs out of 34 identified metabolites	Energy metabolism: Krebs cycle	[25]

Human erythroleukemia cells: K562 and K562/R	Rosemary phenolic extract (carnosol: 226.4 µg/mg; carnosic acid: 151.5 µg/mg); 5, 10 µM	Transcriptomics	Microarray; RT-qPCR	—	289 DEGs in K562; 387 DEGs in K562/R	Cellular movement; cell-to-cell signaling and interaction; free radical scavenging; cell death; NRF2-mediated oxidative stress response; LPS/IL-1-mediated inhibition of RXR function; aryl hydrocarbon receptor signaling; protein synthesis; antigen presentation; cellular function and maintenance	[22]

		Metabolomics (nontargeted)	CE-MS and UPLC-MS	—	121 DEMs in K562; 105 DEMs in K562/R	Aminoacyl-tRNA biosynthesis; glutathione metabolism; arginine and proline metabolism; nitrogen metabolism; glutamate metabolism; urea cycle and metabolism of amino groups	

*Note*: LPS, lipopolysaccharide; IL-1, interleukin-1; RXR, retinoid X receptor; CE-MS, capillary electrophoresis–mass spectrometry; HILIC/UPLC-MS, hydrophilic-interaction ultra-performance liquid chromatography coupled to mass spectrometry; HMD, human metabolome database; ROS, reactive oxygen species; MALDI-TOF/TOF-MS, matrix-assisted laser desorption/ionization-time of flight mass spectrometry; RP, reverse phase; ESI, electrospray ionization; NMR, nuclear magnetic resonance; NRF2, nuclear factor-like. DEG, differentially-expressed gene; DEP, differentially-expressed protein; DEM, differentially-expressed metabolite.

## PC modulation of metabolic pathways and their association with common diseases

**Figure 2:**
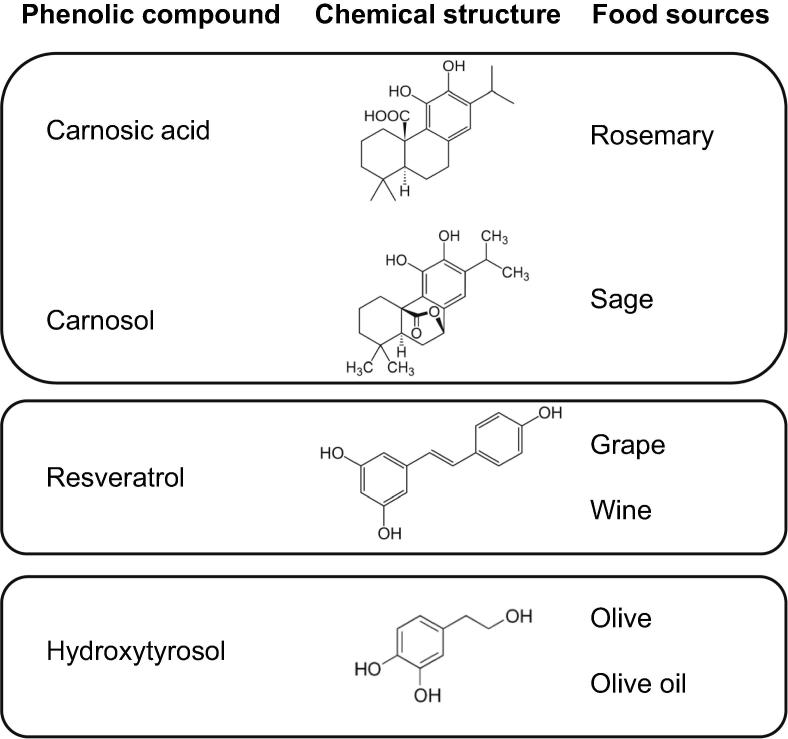
**Chemical structure and main food sources of the phenolic compounds included in this update**

**Figure 3:**
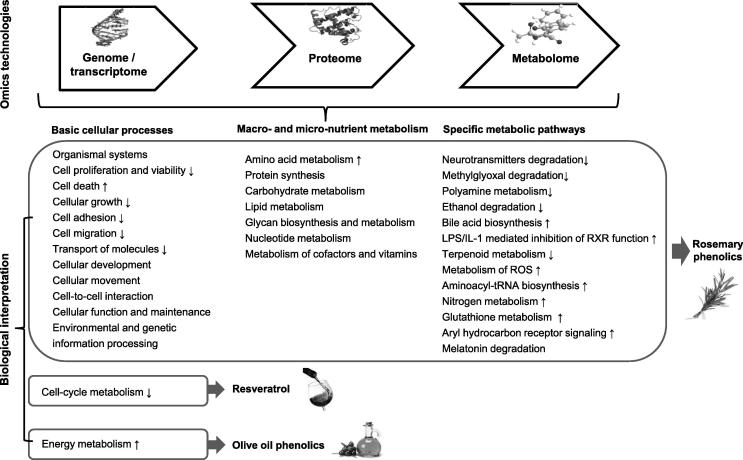
**Metabolic pathways modified by the phenolic compounds reviewed in this update** All the metabolic pathways shown in the figure are modified by rosemary phenolic extract. The increase and decrease in the biological functions are indicated with arrows ↑ and ↓, respectively. Biological functions without arrows indicate that the increase or decrease of these functions is not specified in the original articles.

Information regarding the metabolic pathways supporting the biological role of PCs has been obtained from the metabolomic evaluation after the *in vitro* experiments with rosemary and olive oil phenolics and resveratrol [20], [21], [22], [23], [24], [25], [26]. The chemical structure and food sources of the main PCs analyzed in the present update are represented in Figure 2. Based on the reviewed studies, we concluded that the principal functions modified by these PCs can be classified into (1) basic cellular processes, (2) macro- and micro-nutrient metabolism, and (3) specific metabolic pathways. The cellular processes and pathways that were found to be modulated are listed and classified in Figure 3.

The basic cellular processes modulated by PCs of the present review include: organismal systems, cell proliferation and viability, cellular development, cellular growth, cell death, cell-cycle metabolism, cell adhesion, cell migration, cellular movement, cell-to-cell interaction, cellular function and maintenance, transport of molecules, environmental and genetic information processing, as well as energy metabolism. The macro- and micro-nutrient metabolism modulated by PCs is related to protein, amino acid, lipid, glycan, nucleotide, carbohydrate, cofactors, and vitamins. Only the rosemary phenolic extract but not olive oil phenolic extract and resveratrol can modify macro- and micro-nutrient metabolism and specific metabolic pathways (Figure 3). The specific metabolic pathways modulated are explained in greater detail below.

### Methylglyoxal degradation

Methylglyoxal (MGO) is a highly reactive dicarbonyl compound produced as a by-product of glycolysis and represents the main precursor of advanced glycation end-products that are related to several diseases. Normally, cells are protected against MGO by the glyoxalase system, the main detoxification pathway of MGO [27]. Increased levels of MGO and glyoxalase system dysfunction have been related to several age-related diseases, such as diabetes, CVD, cancer, and neurological disorders [28]. There is evidence supporting that ferulic acid and related PCs can decrease MGO induced-cytotoxicity in reduced glutathione (GSH)-depleted rat hepatocytes by exerting a radical scavenging protective mechanism [29]. These findings are consistent with the reported up-regulation of the gene encoding aldo–keto reductase family 1 member C1 (*AKR1C1*) in the HT-29 colon cancer cell line after the incubation with the phenolic diterpene carnosic acid [20]. AKRs are known as the major enzymes implicated in the reduction of carbonyl substrates that are involved in the synthesis of endogenous compounds as well as xenobiotic detoxification, providing a protection against cellular damage [30].

### Polyamine metabolism

Polyamines such as putrescine, spermidine, and spermine can interact with proteins, DNA, and RNA, and play various roles in cell growth and proliferation. Variations in polyamine levels have been related with certain diseases such as stroke, cancer, renal failure, inflammation, or diabetes [31]. Thus, polyamine function and metabolism is an attractive target for therapeutic intervention, especially for antiproliferative usages [32].

Protective effects against cancer of PCs have been described in HT-29 cells treated with carnosic acid, which are potentially related to polyamine metabolism modulation. This metabolic change is explained by an up-regulation of the gene encoding monoamine oxidase type B (*MAO-B*) that is involved in the enzymatic transformation of *N*-acetylputrescine to 4-acetamidobutanal, thus resulting in the reduced level of *N*-acetylputrescine with carnosic acid treatment [20]. Similarly, Caco-2 cells exposed to a procyanidin-enriched extract showed a significant reduction in two key enzymes of polyamine biosynthesis (ornithine decarboxylase and *S*-adenosylmethionine decarboxylase), leading to a decrease in intracellular polyamines [33]. These results suggest that polyamine pathway could be an important target for PCs as anti-proliferative agents.

### Dopamine, noradrenaline, and serotonin degradation

Dopamine, noradrenaline, and serotonin are neurotransmitters, and their reduction contributes to the occurrence of cognitive decline and neurodegeneration [34]. Specifically, the dopaminergic system as well as abnormal serotonin neurotransmission may be related with the occurrence of Alzheimer’s disease (AD) [35] and Parkinson’s disease (PD) [36]. A foodomic approach has revealed that carnosic acid induces transcriptional regulation of several genes implicated in the degradation of dopamine, noradrenaline, and serotonin, providing new evidence for potential signaling pathways modulated by PCs [20]. In line with these observations, a potential effect of epigallocatechin-3-gallate has been shown against lipopolysaccharide-induced neurotoxicity through reducing inflammatory mediators’ tumor necrosis factor-alpha and nitric oxide and maintaining dopamine levels in midbrain [37].

Another canonical pathway implicated in neurotransmitter metabolism and revealed to be regulated by rosemary phenolics in this update is the tryptophan depletion [20], which has been shown to take part in the development of AD, PD, and Huntington’s disease (HD) [38]. Recent findings have also shown that PCs can inhibit tryptophan breakdown in peripheral blood mononuclear cells [39].

### Melatonin degradation

A relationship has been established between melatonin and aging, neurodegeneration [40], T2DM [41], autism [42], as well as bone status [43]. This pineal hormone has atheroprotective effects [44] and is able to behave as a ‘smart killer’ by modulating apoptosis processes in cancer cells and promoting survival in non-tumor cells [45]. This update shows for first time the association between polyphenols and melatonin degradation pathway at the gene level, after the *in vitro* exposure of cells to rosemary phenolics [20].

### Ethanol degradation

It has been reported that heavy or binge alcohol intake increases the risk of all-cause mortality, whereas moderate consumption, especially of wine and beer, might have cardioprotective effects [46]. A foodomic study has revealed that, enzymes related to ethanol degradation pathway were positively modulated in HT-29 cells after treatment with carnosic acid. In particular, the up-regulation of the gene encoding aldehyde dehydrogenases 3A1 (*ALDH3A1*) could also counteract the formation of aldehydes produced by monoamine oxidases [20]. In line with this study, catechins from black tea partially prevent alterations of oxidative parameters induced by ethanol in old rats [47]. These findings suggest a beneficial effect of PCs on the ethanol degradation pathway.

### Terpenoid metabolism

Terpenoids (also called isoprenoids) and particularly retinoic acid, have been shown to down-regulate proliferation markers and to inhibit tumor growth, angiogenesis, and metastasis [48]. Additionally, direct actions of retinoids have been reported in insulin secretion through pancreatic β-cell function [49], and the maintenance of immunity, reproduction, and embryonic development [50]. Early work has also shown that the level of long-chain isoprenoid dolichol is decreased, whereas the level of dolichyl phosphate and ubiquinone is increased in brains of AD patients [51]. Moreover, as the important regulators of macrophages [52], retinoid X receptors (RXR) might inhibit the initial inflammatory process preceding the atherogenesis [53]. Pathway analysis indicates that the metabolism of terpenoid pathway could be significantly repressed in HT-29 cells treated with carnosic acid by the reduced expression of genes encoding alcohol dehydrogenase 1C (*ADH1C*), endothelin 1 (*EDN1*), nuclear receptor subfamily 4 group A member 1 (*NR4A1*), and prolactin receptor (*PRLR*) [20], suggesting an anticancer protective effect of carnosic acid. Along the same line, a cancer prevention effect has been reported after a long term consumption of a green tea phenolic extract in rats with induced colon carcinoma, due to the increased levels of RXRα [54].

### GSH metabolism

GSH is considered to be one of the most important scavengers of reactive oxygen species (ROS), which has been involved in the progression of a number of aging diseases, such as cancer, CVD, and neurodegenerative diseases [55]. GSH depletion has been described as a common process of apoptotic cell death initiated by various stimuli [56]. Thus, GSH modulation has been thoroughly investigated for potential anticancer treatments.

Several metabolomic studies in leukemia and colon cancer cell lines have demonstrated that rosemary phenolics increase GSH levels with a rise of the GSH/glutathione disulfide (GSSG) ratio [20], [22]. Combined with the transcriptomic data, functional analysis underscored an activation of ROS metabolism and expression modification of several genes related to oxidative degradation pathways in both cell lines. These results suggested that rosemary phenolics could enhance the detoxifying cellular capabilities, which is necessary for cancer growth inhibition. It has also been suggested that PCs modify intracellular GSH concentrations through modulating detoxification processes and protein glutathionylation, as well as adjusting the redox switching of protein functions [57].

### Aminoacyl-tRNA biosynthesis

Aminoacyl-tRNA synthetases (ARSs) are essential for the first step in protein synthesis. It has been reported that defects in ARS functions may contribute to several diseases such as cancer [58]. A metabolomic approach for leukemia cells treated with rosemary PCs showed an increased expression of the gene encoding valyl-tRNA synthetase (*VARS*), which is related to aminoacyl-tRNA biosynthesis pathway. In addition, the authors also detected changes in the amount of related metabolites, in particular some amino acids such as Met, Leu, Glu, Tyr, Lys, and Phe [22]. Along the same line, an increase in aminoacyl-tRNA content in osteoblastic MC3T3-E1 cells was observed due to the action of the isoflavone genistein, which is involved in the activation of ARSs [59].

### Aryl hydrocarbon receptor signaling

The activation of aryl hydrocarbon receptor (AhR) has been shown to reduce immune responses, leading to suppression of autoimmune diseases [60], and possibly reduce inflammation associated with Crohn’s disease as well [61]. An abnormal AhR function has also been associated with cancer [62]. The AhR signaling, which is associated with the transcription of genes encoding metabolizing enzymes and antioxidant proteins, was enhanced in leukemia cells in response to rosemary PCs [22]. To screen for natural AhR agonists in foods, Amakura et al. assessed multiple food materials and found that cassia seed and rosemary extracts exhibited high AhR activity [63].

### Bile acid biosynthesis

Beside the well-established role of bile acids in cholesterol homeostasis and lipid absorption, Kuipers et al. have also demonstrated glucose-lowering actions of bile acids in insulin-resistant states and T2DM [64]. Additionally, high concentrations of deoxycholic acid have been shown to induce cell proliferation, causing hyperproliferation in the colorectal mucosa, which is an early stage in colorectal cancer [65]. Administration of 0.5% apple phenolic extract in rats leads to an up-regulation of the expression of farnesoid X receptor that takes part in the bile acid biosynthesis and consequently improved blood cholesterol levels [66]. Investigation on colon cancer HT-29 cells treated with carnosic acid has revealed that the phenolic carnosic acid from rosemary can modulate the biological process of bile acid biosynthesis [20].

## Translational perspective of PCs in disease prevention

After in-depth examination of the pathways modulated by the PCs studied, the next step is to analyze the illnesses and disorders involving these pathways. Based on the integrated data, it seems that the PCs could have an essential role mainly in the prevention of neurodegenerative disorders, T2DM, cancer, and CVD (Table 2**)**. In particular, carnosic acid and carnosol, the main PCs present in rosemary, appear to be implicated in most of the metabolic pathways analyzed in Table 2.

**Table 2:** **Metabolic pathways modified by PCs reviewed and their association with common diseases**

**Metabolic pathway**	**Neuro-degenerative disorders**	**Neurological diseases**	**T2 DM**	**CVDs**	**Cancer**	**Digestive diseases**	**Reproduction disorders**	**Renal disorders**	**Bone status**	**AIDs**
Neurotransmitter degradation	[35], [36]									

Ethanol degradation				[46]						

Melatonin	[40]	[42]	[41]	[44]	[45]				[43]	

Methylglyoxal degradation			[28]	[28]	[28]					

Polyamine metabolism			[31]	[31]	[32]			[31]		

Terpenoid metabolism	[51]		[49]	[52], [53]	[48]		[50]			

Tryptophan degradation	[38]									

Glutathione metabolism				[55]	[55]					

Aminoacyl-tRNA biosynthesis					[58]					

Aryl hydrocarbon receptor signaling					[62]	[61]				[60]

Bile acid biosynthesis			[64]		[65]					

*Note*: Neuro-degenerative disorders include Alzheimer’s disease, Parkinson’s disease, and Huntington's disease. Cardio-vascular diseases include inflammatory response, oxidation response, and atherosclerosis. PC, phenolic compound; T2DM, type 2 diabetes mellitus; CVD, cardiovascular disease; AID, autoimmune disease.

Resveratrol is mainly involved in energy metabolism regulated by a peroxisome proliferator-activated receptor [67], [68], and this pathway has been related to the nervous system, cancer, diabetes, and neurodegenerative disorders, such as AD [69], [70]. Extra virgin olive oil phenolic extract is mostly associated with cell-cycle metabolism. Dysregulation of this process has been related with the etiology of major chronic pathologies such as cancer, atherosclerosis, inflammation, and neurodegenerative disorders such as AD, HD, and PD [71].

Plant-derived food, such as fruits, vegetables, aromatic herbs or olive oil, contains a diverse group of PCs with many potential disease prevention effects. Occidentals consume more than 1 g/day of PCs and even close to 3 g/day if fiber-bound PCs are taken into account [72]. However, only less than 5% is directly absorbed and metabolized by the body [72]. Once absorbed, PCs are extensively altered during first-pass metabolism so that, typically, the molecular forms reaching the peripheral circulation and tissues are different from those present in foods. The resulting metabolites have different molecular structures (mainly sulphated and glucuronidated forms of the parent aglycone) and consequently their biological activities in the target organs can differ.So, under physiological situation, plasma and tissues are not exposed to PCs in the aglycone forms [73]. Therefore, future metabolomic cell-based studies should be performed to test the conjugated forms of PCs found in plasma with the concentration range of 0.1–10 μM, the maximum plasma concentrations detected after normal consumption of PC-rich foods [73]. Thus, human data concerning bioavailability and metabolism of PCs are of great value for future studies focusing on defining biological activities of PCs.

Despite the promising role of PCs in the improvement of cellular functions and pathways, evidence is not strong enough to suggest the optimal intake doses. The underlying mechanisms of PCs in chronic disease prevention are still poorly understood. Therefore, the recommendation is to eat a variety of vegetables, fruits, and other plant-derived food containing different types of PCs, which presumably provides the best protection.

## Future developments

The metabolic data available up till now offer promising knowledge about the role of dietary PCs in preventing and/or protecting against various diseases through the modification of several metabolic pathways. Thus, metabolomic profiling techniques used in cell-based studies provide unprecedented opportunities to evaluate the mechanisms-of-action for PCs, with complementary data of human studies. Metabolomic data also provide a more realistic understanding of the role of PCs in disease recognition/prevention. According to Lee [74], the *in vitro* metabolomic model can be employed to fully elucidate whether the *in vivo* observations actually have molecular basis.

In conclusion, assessing the biological effects of specific PCs by *in vitro* metabolomic approaches has contributed to better definition of a metabolomic signature and enhanced understanding of disease mechanisms in response to PCs exposure. Moreover, the reviewed metabolomic data have provided the possible clinical impact of PCs on common diseases, concluding that PCs are mainly implicated in neurodegenerative diseases, T2DM, cancer, and CVDs. Despite its analytical capacity, the utilization of metabolomic approach in the study of disease prevention by PCs is still in its infancy. Therefore, new and more refined metabolomic approaches testing other types of PCs are needed to finally elucidate the underlying mechanisms of specific PCs in human physiological and pathological processes.

## Competing interests

The authors have declared no competing interests.
